# Analyzing the Accuracy of Digital Sizing on Long-Leg Alignment X-rays by Using a 1-Inch Ball Bearing: A Cheap and Effective Method

**DOI:** 10.7759/cureus.55735

**Published:** 2024-03-07

**Authors:** Trey D VanAken, Daniel Joiner, Lauryn Boggs, Andrew Robinson, Nahel Haji, Rahul Vaidya

**Affiliations:** 1 Department of Orthopaedic Surgery, Wayne State University Detroit Medical Center, Detroit, USA

**Keywords:** long-leg radiographs, digital planning, ball bearing, ct scanogram, computerized tomography, long leg standing alignment films, digital sizing, digital templating

## Abstract

Background and objective

Sizing on digital films is important for implants and planning deformity correction. CT is the most accurate digital measurement method. We use a 1-inch ball bearing (cost: $1) to size our long-leg standing films (LLSFs) when planning deformity correction. In this study, we aimed to assess the accuracy of digital measurements calibrated by this method.

Methods

We conducted An IRB-approved study involving 25 patients having both an LLSF with a 1-inch ball bearing taped to the inner mid-thigh and a CT scanogram. The longest distance in the axial cut of the bilateral ankle, knee, and femoral heads of the CT images were compared to the same anatomic locations on LLSFs calibrated with the ball bearing using the online digital planning software DetroitBonesetter (DBS) and measurements from our Picture Archiving Communication Software (PACS). Five observers performed each measurement.

Results

The average measurement differences between the gold standard CT scan and LLSFs calibrated with DBS were as follows: 0.110 ± 0.432 mm (femoral head); 2.173 ± 0.0619 mm (knee); and 3.671 ± 0.30 mm (ankle). In PACS, they were as follows: 5.470 ± 0.381 mm (femoral head); 6.248 ± 0.712 mm (knee); and 1.806 ± 0.548 mm (ankle). The intraclass correlation coefficient for 600 measurements by five observers was 0.972.

Conclusions

The $1 ball-bearing sizing on DBS using LLSFs provides accuracy to <1 mm for the femoral head, 2 mm at the knee, and 3.7 mm at the ankle. It was significantly better than the PACS system for both the femoral head and knee (<0.001), while PACS was better at the ankle (<0.001).

## Introduction

Preoperative planning in orthopedic surgery has two parts: the logistics and the surgical tactic. While a plan can be roughly formulated on paper, in certain situations, such as in arthroplasty or lower limb deformity correction, the measurements must be accurate regarding the size of an implant, opening or closing wedge osteotomy, as well as correction of leg-length discrepancy (LLD) [1-5]. Sizing X-rays whether on film or digital imaging has been associated with magnification discrepancies [3-6]. A study by Ravi et al. found that there was a 21-31% magnification error in plain radiographs and Rampersaud et al. noted up to 40% magnification error when compared to CT imaging. They noted that the use of digital sizing helped to significantly reduce these error rates [7,8].

CT is considered the most accurate method of digital measurement, but it is not in routine use due to cost constraints and large amounts of radiation exposure, and the fact that orthopedic surgeons are used to planning using 2D technology, only using 3D in special circumstances [9-13]. Measurements on digital Picture Archiving and Communication System (PACS) are often wrong and the use of reference sizers has been advocated to improve the precision of digital sizing. Several studies have described the accuracy of various types of reference markers in plain film imaging [14-21]. Accuracy is also necessary for the planning and execution of deformity correction, which are often planned on long-leg standing films (LLSFs). Spherical reference balls have been used in the preoperative planning of high tibial osteotomies using expensive software and calibration markers. However, there are no studies in the literature validating these measurements [22-24].

We propose the use of spherical 1-inch truck ball bearings, as cost-effective, precise reference markers for preoperative digital templating. These truck ball bearings have remarkably precise sizing as they carry tons of pounds traveling at high rates of speed and cost approximately $1 each. We recommend the use of these ball-bearing sizers on long-leg alignment views using a free online digital planning software: DetroitBonesetter (DBS) (https://detroitbonesetter.com, Detroit, MI) [25] as a cheap and accurate method for preoperative digital sizing. This study aims to examine the accuracy of this method of collecting digital measurements from LLSFs and compare it to PACS sizing of the LLSFs and CT images.

## Materials and methods

As mentioned above, many studies in the literature have described the accuracy of various types of reference markers in plain film imaging (Table 1).

**Table 1:** A summary of prior studies on various reference markers used for standardization of X-ray sizing, their methods, cost, and effectiveness THA: total hip arthroplasty; TKA: total knee arthroplasty; CT: computed tomography

Reference #	Digital marker used	Marker utilization	Estimated cost of marker	Effectiveness
[6]	25.4 mm metallic sphere	THA: taped to the side of the hip at the greater trochanter; TKA: taped to the midline anterior thigh just proximal or at the level of the patella	Varies: $20-$70	In 93% of THAs and 98.5% of TKAs, preoperative templating was within 1 size of the final implant
[14]	30 mm calibrated ball	THA: between the legs as close to the joint as possible; TKA: at the level of the joint line	Varies: can be $100 or more	Reference ball improved precision of preoperative planning, more in TKAs (>90% within 1 size) than THAs
[15]	10 pence coin	Taped to the lateral aspect of the hip at the greater trochanter	14 cents	Compared with other templating methods used, 10 pence coin was most accurate with only 0.9% undersizing but was not as reproducible
[16]	Ball bearing	Taped to the medial aspect of the knee at the joint line		Template was accurate to within 1 implant size, 100% of the time
[17]	25 mm metallic ball	THA: taped to the side of the hip at greater trochanter; TKA: taped to midline anterior thigh at the level of the patella	Varies: $20-$70	In 73% of THAs and 92% of TKAs, digital templating was within 1 size of the actual implant used
[18]	30 mm spherical scaling ball	The ball placed adjacent to the joint line	~$30	Allowing ± 1 implant size, templated size was almost 100% accurate for TKA
[19]	Ball bearing	Ball bearing in plastic pipe put between the patient’s thighs		The size of the marker was measured for consistency and was very accurate with a mean of 19.95 and a median and mode of 20 mm
[20]	Virtual disc	A planar disc placed on the radiographic cassette to account for the expected magnification	Variable but CT scan was used, and hence at least 5x more expensive than X-ray	The disc proved more accurate and reliable than the sphere method when using CT
[21]	10 pence coin	Taped to the lateral hip at the greater trochanter	14 cents	No significant difference in the accuracy of templated vs. implanted prosthesis with the magnification marker

**Figure 1:**
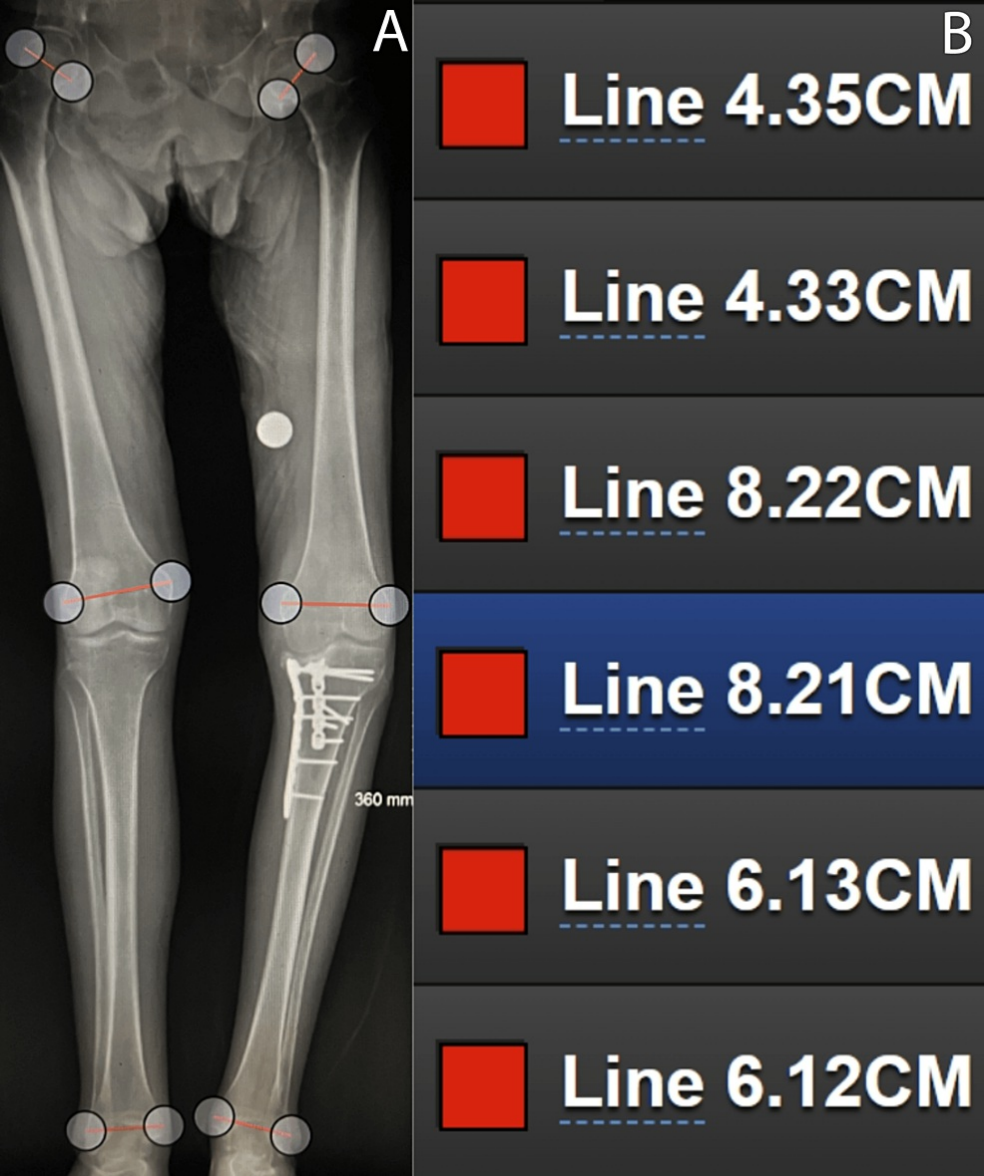
DetroitBonesetter measurements Full set of measurements using LLSF and DBS by one of our observers (A). The measurements shown are grouped by joint and listed from top to bottom as femoral head, knee, and ankle measurements (B) LLSF: long-leg standing film; DBS: DetroitBonesetter

This was an IRB-approved retrospective study involving 25 patients. Before capturing LLSFs, a 1-inch (25 mm) truck ball bearing was taped to the inner mid-thigh by our radiology technicians in a standard manner. Originally, the ball-bearing placement was attempted at the level of the knee. The overlap between radiopaque bone and ball bearing made it difficult to utilize the auto-sizing feature within DBS. This feature greatly improved the reproducibility of the measurements. Greater soft tissue coverage over the thigh enabled sufficient separation between the ball bearing and osseous structures to allow for measurement. Twenty-five patients were found to have undergone both this LLSF with ball bearing and a CT scanogram for axial measurements. The LLSFs were made by using a standard three-shot protocol and stitched by the PACS system (done automatically), which is more accurate than knee-centered images [26]. For these 25 patients, measurements of the longest distance in the axial cut of the bilateral ankle, knee, and femoral head of the CT scanogram images by using PACS were completed. The widest point of the same three anatomic locations was measured bilaterally, on the same patient's LLSF, by using the freely available online digital planning software DBS. Images of a set of measurements collected using DBS are presented in Figure 1.

**Figure 2:**
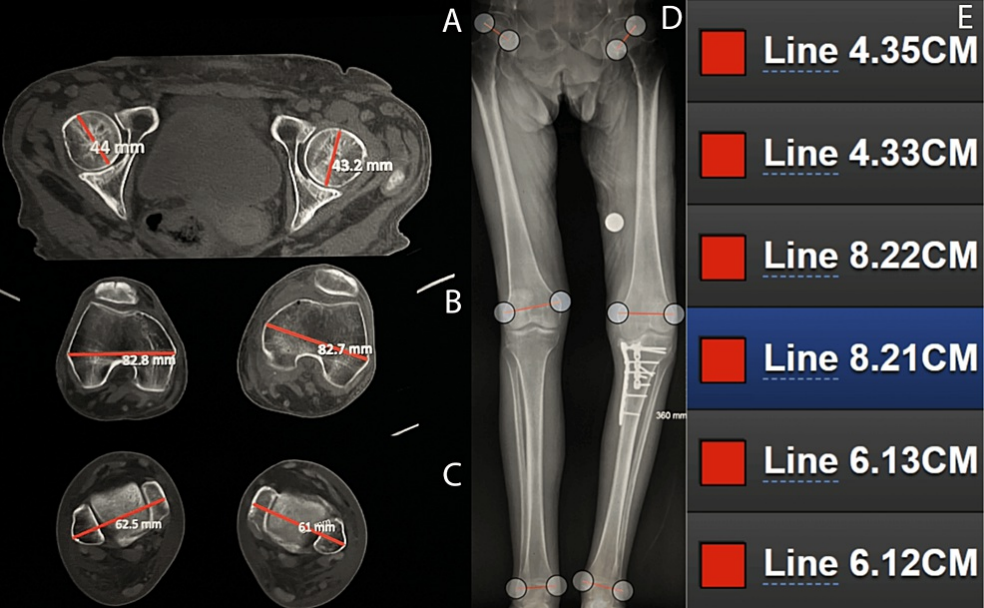
CT scanogram vs. DBS measurements Images showing a set of femoral head (A), knee (B), and ankle (C) measurements performed on CT images with the PACS software as compared to measurements of the same anatomical sites on the same patient using an LLSF and DBS (D). The measurements shown are grouped by joint and listed from top to bottom as femoral head, knee, and ankle measurements (E). This comparison highlights the precision achieved when measured using the zoom function in DBS CT: computed tomography; LLSF: long-leg standing film; DBS: DetroitBonesetter; PACS: Picture Archiving and Communication Software

The results were compared to CT scanogram measurements made of the same patients’ limbs (Figure 2).

A paired t-test and nonparametric Wilcoxon test were conducted as normal distribution was not fully met. Five different observers measured each point, and an average number was determined for each of the 600 measurements. The intraclass correlation coefficient was calculated between the observers. A standardized measuring protocol was followed by all observers, which is detailed in Table 2.

**Table 2:** LLSF measurement protocol used by all observers in this study while conducting measurements in DetroitBonesetter* *[25] Measurement protocol that was developed and utilized throughout this study LLSF: long-leg standing film

DetroitBonesetter standing orthogram measurement protocol
Select the "X-ray" button to import long-leg standing film from the computer into Bonesetter
Remove confidential information if needed, then press "Next"
Select "Automatically with Bonesetter Sizer" as the scaling method
In the upper left corner, find the dropdown tab with the label "Sizer"
Select "25 mm Scale" as the size from the dropdown menu in the left corner
Leave the Magnification Corrections tab set at "None"
Zoom in on ball bearing and then click on the ball bearing to highlight the radiopaque area that the ball bearing creates
Ensure the entirety of the ball bearing is highlighted; often the images have a lighter ring surrounding a darker core; highlight both the core and outer ring by clicking on the outer ring directly
When satisfied with highlighting, click the "Import" button
Select the "X-ray" tab and lock the image by clicking the image of an unlocked padlock until it appears locked
Now that the image has been properly scaled and locked, you are ready to take your measurements
Zoom image to 500%; in the upper right corner, there is a zoom percentage box you can type this value in
Click the "Line" button and begin measuring the widest point of the bilateral hips, femoral condyles, and ankle joints
Place lines for hips through the center and widest part of the spherical ball joint
Place lines for knees at the widest point of the femoral condyles, roughly parallel to naturally forming joint space
Place lines for ankles at the widest point of the joint, roughly parallel to the top of the calcaneus bone and naturally forming joint space
Once finished, press "Save" in the upper right toolbar, to save your file

## Results

CT scanogram and LLSF

Data analysis revealed that the average differences in measurements using CT scanogram versus those using LLSFs in DBS were as follows: for the femoral head: 0.110 ± 0.432 mm (p=0.399); for the knee: 2.173 ± 0.0619 mm (p<0.001); for the ankle: 3.671 ± 0.30 mm (p<0.002). Given that the p-value was greater than 0.05 for the femoral head measurements, the mean difference calculated in this location is not statistically significant. However, the average differences calculated at the knee and the ankle joint were statistically significant, as shown in Table 3. The intraclass correlation coefficient was calculated to be 0.972 for average measures, and 0.902 for single measures, among the individual observers (p<0.001). This is indicative of the reproducibility of the DBS ball bearing, digital templating method, as values approaching 1 indicate a high degree of correlation among observers.

**Table 3:** Study results All CT images were collected using our institution's PACS, while all LLSF images were measured using DetroitBonesetter CT: computed tomography; LLSF: long-leg standing film; PACS: Picture Archiving and Communication Software

PACS CT measurements vs. DetroitBonesetter LLSF measurements					
	Mean (mm)	Mean difference (mm)	Standard error (mm)	Standard deviation (mm)	P-value
CT femoral head	45.966		0.296	4.017	
LLSF femoral head	45.856		0.489	6.645	
CT vs. LLSF femoral head		0.110	0.432	5.865	0.399
	Mean (mm)	Mean difference (mm)	Standard error (mm)	Standard deviation (mm)	P-value
CT knee	83.478		0.464	6.036	
LLSF knee	81.31		0.836	10.871	
CT vs. LLSF knee		2.168	0.619	8.049	<0.001
	Mean (mm)	Mean difference (mm)	Standard error (mm)	Standard Deviation (mm)	P-value
CT ankle	64.453		0.424	5.674	
LLSF ankle	60.78		0.419	5.608	
CT vs. LLSF ankle		3.673	0.30	4.019	<0.002

PACS vs. DBS

When measuring the same LLSFs using the PACS and comparing them to CT scanogram measurements collected with PACS, the average differences found were as follows: for the femoral head: 5.470 ± 0.381 mm; for the knee: 6.248 ± 0.712 mm; and for the ankle: 1.806 ± 0.548 mm. The DBS ball bearing method for measuring LLSFs was significantly better than LLSF measurements collected using the digital PACS system concerning the femoral head (p<0.001) and knee (p<0.001), while the PACS system was better at ankle measurement (p<0.001) (Table 4).

**Table 4:** PACS measurements comparison Table demonstrating the results of comparing PACS measurement of both CT imaging and LLSF imaging at the femoral head, knee, and ankle for all patients CT: computed tomography; LLSF: long-leg standing film; PACS: Picture Archiving and Communication Software

PACS CT measurements vs. PACS LLSF measurements					
	Mean (mm)	Mean difference (mm)	Standard error (mm)	Standard deviation (mm)	P-value
CT femoral head	45.988		0.692	4.793	
LLSF femoral head	51.458		0.568	3.932	
CT vs. LLSF femoral head		5.470	0.381	2.645	<0.001
	Mean (mm)	Mean difference (mm)	Standard error (mm)	Standard deviation (mm)	P-value
CT knee	83.519		0.909	5.962	
LLSF knee	89.767		1.281	8.40	
CT vs. LLSF knee		6.248	0.712	4.670	<0.001
	Mean (mm)	Mean difference (mm)	Standard error (mm)	Standard deviation (mm)	P-value
CT ankle	64.343		0.819	5.617	
LLSF ankle	66.149		0.852	5.838	
CT vs. LLSF ankle		1.806	0.548	3.762	<0.001

## Discussion

**Figure 3:**
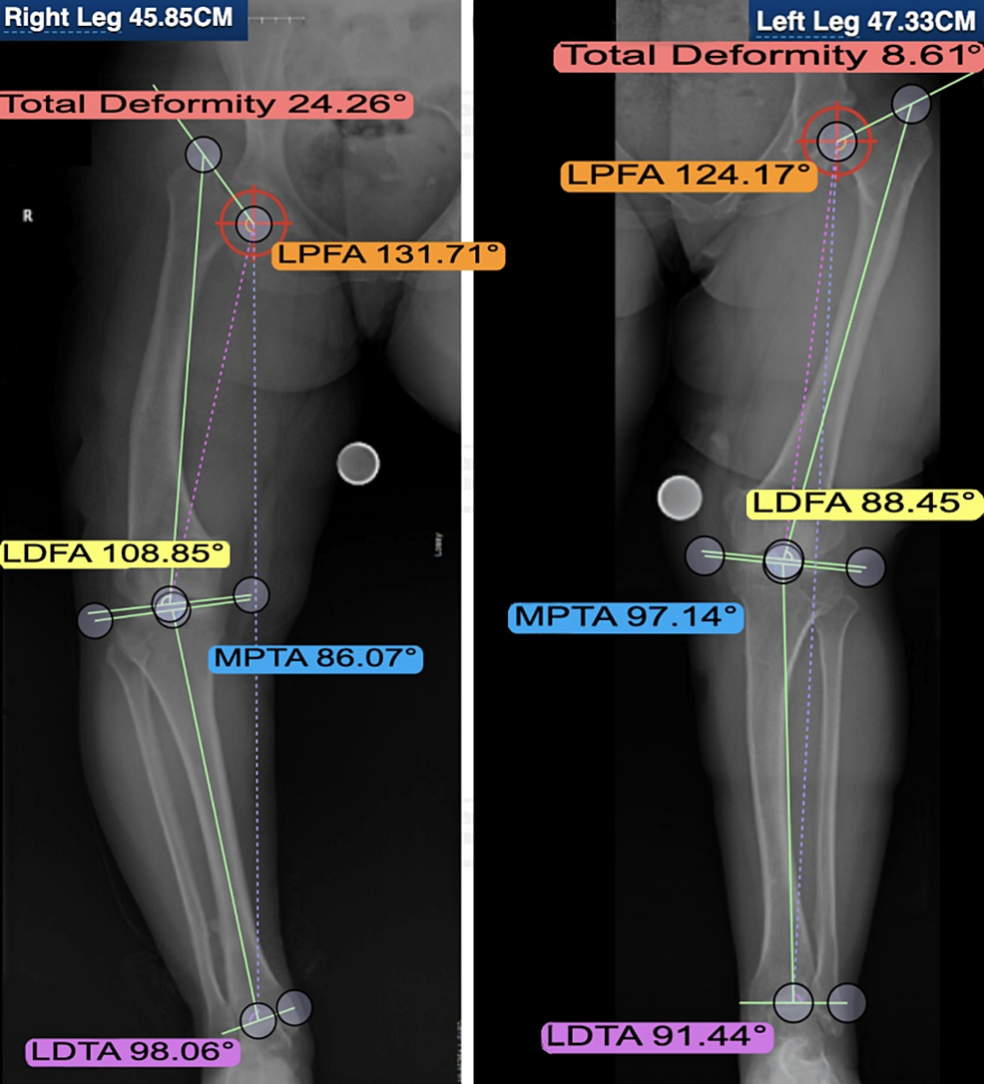
Preoperative application of DetroitBonesetter Pre-operative imaging of a patient whose surgery was planned using DetroitBonesetter software LPFA: lateral proximal femoral angle; LDFA: lateral distal femoral angle; MPTA: medial proximal tibial angle; LDTA: lateral distal tibial angle

**Figure 4:**
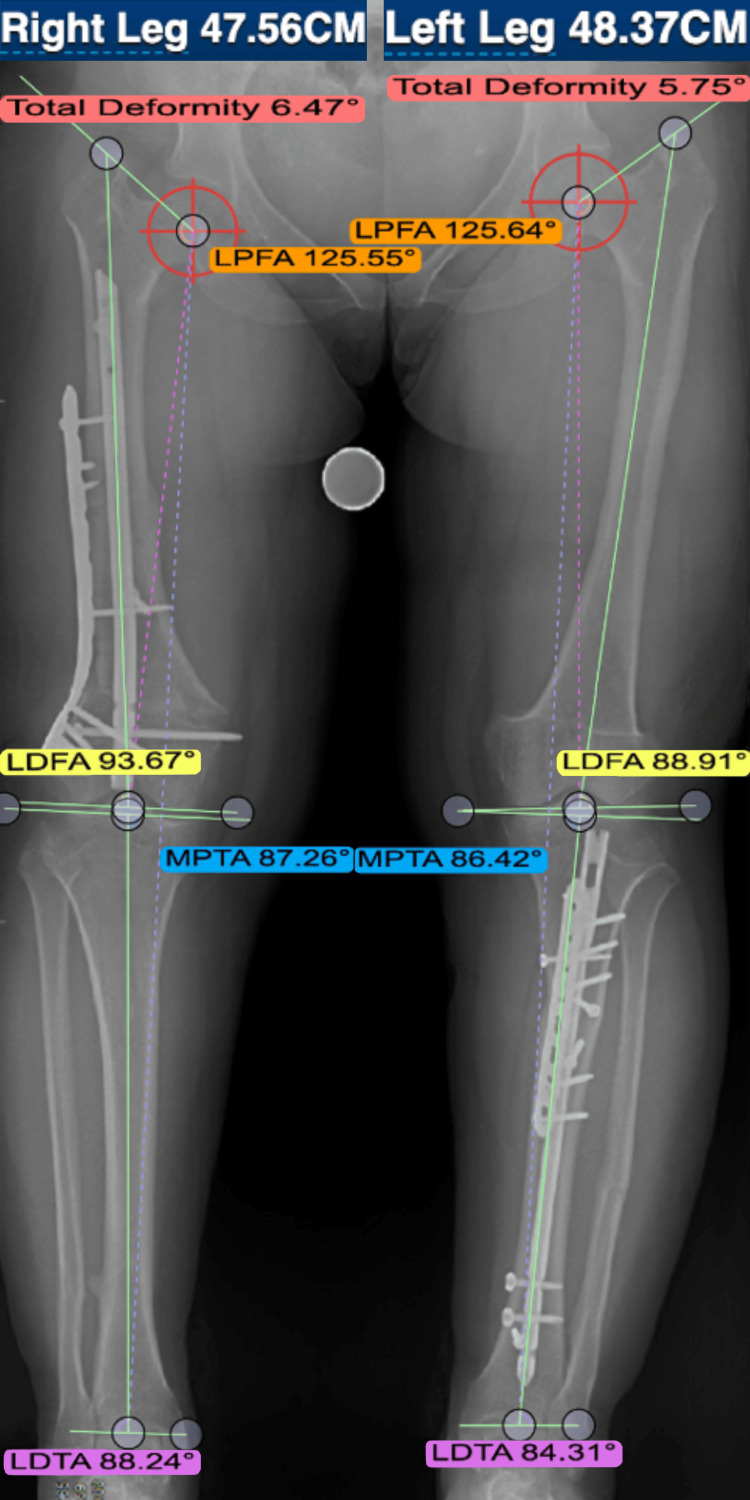
Postoperative application of DetroitBonesetter Postoperative imaging of a patient whose surgery was planned using DetroitBonesetter software, demonstrating the correction of leg-length discrepancy and coronal alignment LPFA: lateral proximal femoral angle; LDFA: lateral distal femoral angle; MPTA: medial proximal tibial angle; LDTA: lateral distal tibial angle

As the use of digital templating is on the rise in the field of orthopedics, there is an increased need to develop reproducible, cost-effective methods for accurately conducting this form of preoperative planning. our findings showed that the use of a low-cost 1-inch ball bearing with DBS for digital templating of X-rays is accurate and reproducible when compared to the gold standard, CT imaging. While this study quantifies the accuracy of digital sizing using DBS, this method has been proven in practice with actual surgical planning, as shown in Figure 3 and Figure 4, which demonstrate pre and postoperative imaging of a patient with achondroplasia, a windswept deformity, and LLD, successfully treated with three osteotomies to correct the coronal alignment and LLD to within 1 cm.

CT imaging is universally regarded as the gold standard for the assessment of linear bone geometry. Studies on linear CT measurements using cadaveric specimens have reported a mean error in the range of 0.06-0.8 mm, varying based on anatomic location and CT manufacturer [9-13]. When comparing the accuracy of CT to MRI in determining the inter-marker distance of two markers placed on cadaveric knees, CT had a 99% accuracy while MRI only had a 97.5% accuracy [12]. A study by Rubin et al. compared measurements of femoral geometry taken using CT and X-ray, comparing them to measurements of the same femurs using calipers. They found a mean measurement difference of 2.4 ± 1.4 mm for X-ray and 0.8 ± 0.7 mm for CT [13]. Measurements of bone geometry collected using these modalities are linear or angular, with linear measurements having the lowest intra-observer variability among the two types, highlighting their reproducibility [22]. Linear CT measurements of bone geometry are the most accurate and they are reproducible, which is why it was chosen as the gold standard for comparison in this study.

Prior research regarding digital reference sizers has described the use of a coin piece and spherical ball bearing to decrease magnification discrepancies in plain films, with the most accurate marker being the spherical ball bearing [14-16,23-28]. Studies involving a coin as the reference marker demonstrated that this method lacks reproducibility, likely due to the variation in size of the coin from rotational positioning in the radiograph [7,16,17]. Kulkarni et al. described using this type of marker with increased accuracy; however, the method used included CT scans, and trigonometry functions leading to greatly increased cost and complexity [26]. One issue with current spherical reference balls is that they can be unnecessarily expensive, and often require the use of costly external software for accurate digital templating. This study addresses these shortcomings with a cheap yet accurate semi-truck ball bearing and free-to-use online software (DBS), demonstrating that our method is a cheap, practical alternative for getting accurate results. Prior calibration studies have been done on simple X-rays for total hip arthroplasty which does not readily translate into LLSFs. Carlson et al. reported that when using DBS for templating of total hip arthroplasty components, there was a significant difference between templated and implanted acetabular components with a positive measurement bias of 3.6 mm [29]. It was later found that the authors mistakenly utilized a reference sizer with a diameter of 30 mm; however, it was recorded as 25 mm in diameter. This size discrepancy would account for the positive measurement bias that was reported in their results as DBS was set to calibrate image size based on the use of a 25 mm reference sizer.

The methods used in this study are relevant, as they are applicable on a wide scale, requiring access only to plain LLSF imaging, cheap ball bearings, a computer, and the internet to replicate. The five individuals conducting measurements in this study had a range of experience measuring radiographic images (one fourth-year orthopedic surgery resident, two third-year medical students, one second-year medical student, and one undergraduate research student) and still reported an intraclass correlation coefficient greater than 0.9 for average measurements and single measurements. Hence, this study demonstrates that the DBS ball-bearing method for measuring LLSFs is reproducible by measurers of various skill levels while still yielding accurate results.

This study has a few limitations. The long-leg standing X-rays were performed by several technicians but in a standard manner. Although every attempt was made to make sure both legs were straight and aligned with the kneecaps placed forward, there were small variances. Another difficulty pertained to situations when there was hardware traffic in LLSF imaging, which sometimes made it hard to measure. The location of the ball was always in the upper leg at the midportion, but different thickness legs could result in variances of slightly anterior or posterior placement of the marker to the bone. Even with these limitations, the results demonstrated for both the knee and ankle are statistically significant and highly correlated, indicating reproducibility. The solution for the lower leg may be to place a second marker along the ankle or the mid-tibia, for which we recorded the greatest average variance.

## Conclusions

While CT is still the gold standard for digital sizing, our technique using DBS to size LLSFs with a 1-inch ball bearing reference marker provides accuracy to <1 mm for the femoral head, 2 mm at the knee, and 3.7 mm at the ankle. Placing the ball bearing close to the area of deformity or placing a second ball next to the tibia as well as the femur may correct the error at the ankle. Our findings show that the described method of digital templating is a cheap, accurate, and reproducible technique for preoperative planning in deformity surgery. Employing this method of digital templating will be especially beneficial in countries/regions with medical resource scarcity, as it will provide an accurate technique for perioperative planning of orthopedic procedures in a highly cost-effective manner.
